# Women during the war - stress, resilience and self-efficacy during the Russian-Ukrainian war (May 2022) among women from Ukraine, Poland, Slovakia and Romania

**DOI:** 10.1192/j.eurpsy.2023.1350

**Published:** 2023-07-19

**Authors:** A. Piotrowski, O. Boe, E. Sygit-Kowalkowska, I. Petrovska, A. Predoiu, R. Predoiu, K. Görner, S. Rawat, R. Makarowski

**Affiliations:** 1 Institute of Psychology, University of Gdansk, Gdańsk, Poland; 2 Department of Organisation, Leadership and Management, Inland School of Business and Social Sciences, Rena, Norway; 3 Departament of Psychology, Kazimierz Wielki University in Bydgoszcz, Bydgoszcz, Poland; 4 Department of Psychology, Ivan Franko National University of Lviv, Lviv, Ukraine; 5 Faculty of Physical Education and Sport, National University of Physical Education and Sports, Bucharest, Romania; 6 Faculty of Sports, University of Presov, Presov, Slovakia; 7 Faculty of Management, Symbiosis International, Pune, India; 8 Faculty of Administration and Social Science, Academy of Applied Medical and Social Sciences in Elbląg, Elbląg, Poland

## Abstract

**Introduction:**

The sudden and large-scale Russian invasion of Ukraine has caused significant stress not only in Ukrainians but also in citizens of countries which have received the largest numbers of refugees.

**Objectives:**

The aim of the current study was to identify stress, resilience, and self-efficacy levels, as well as to examine the relationships between these variables, in a sample of women from Ukraine, Poland, Slovakia, and Romania during the third month of Russian aggression on Ukraine.

**Methods:**

The study involved measuring a sample of Ukrainian (N = 82), Polish (N = 102), Slovak (N = 79), and Romanian (N = 42) women using the *Perception of Stress Questionnaire,* the *Brief Resilience Scale,* and the *Generalized Self-Efficacy Scale* in May 2022.

**Results:**

The results showed that during the third month of Russian aggression on Ukraine, stress levels and its components (emotional tension, external stress, and intrapsychic stress) were the highest among Ukrainian women and the lowest among Polish women. The sense of self-efficacy was the lowest among Ukrainian women and highest among Polish women, while resilience was the lowest among Ukrainian women and the highest among Slovak women.

**Conclusions:**

Women from Ukraine reported being in the worst mental state compared to the rest of the sample. Moreover, a path analysis of the measured variables points to a multifaceted relationship between stress, resilience, and self-efficacy among women from Ukraine, Poland, Slovakia, and Romania during the third month of the Russian invasion.

**Disclosure of Interest:**

None Declared

